# Information Provided by Breeders and Referring Veterinarians about the Presence and Meaning of a Murmur to Owners of Newly Purchased Puppies with a Later Confirmed Congenital Heart Disease

**DOI:** 10.3390/vetsci9120678

**Published:** 2022-12-06

**Authors:** Vicky R. Vos, Viktor Szatmári

**Affiliations:** Department of Clinical Sciences, Faculty of Veterinary Medicine, Utrecht University, Yalelaan 108, 3584 CM Utrecht, The Netherlands

**Keywords:** auscultation, dogs, echocardiography, screening

## Abstract

**Simple Summary:**

The first veterinary health check in puppies typically takes place at 6 weeks of age and includes listening to the heart. A heart murmur at this age can be innocent, but it can also indicate the presence of a congenital heart disease. Congenital heart diseases rarely cause clinical signs at this age, but they might lead to severe problems and even death months to years later. The best way to find out what exactly a murmur means is to refer the puppy to a veterinary cardiology specialist for an echocardiogram, preferably before the puppy is sold to a new owner. Breeders usually sell their puppies at 8–10 weeks of age, i.e., weeks after the first veterinary screening. In the present study, all owners whose dogs were diagnosed with a congenital heart disease at the authors’ cardiology service, were asked to fil in a questionnaire. Of the 60 owners, 77% did not know about their dogs’ murmur when they bought them. However, 72% of the owners would not have bought that puppy if they had known about the possible congenital heart disease. We conclude that most owners would refrain from buying a puppy with a hidden heart disease, but, in most cases, they were not informed.

**Abstract:**

**Background**: A recent study revealed that only 10% of puppies diagnosed with a congenital heart disease were referred for murmur investigation to a veterinary cardiology specialist while the puppies were in the breeders’ possession. Whether the new owners had been informed about the presence of a murmur before purchasing a puppy was not investigated. **Methods**: New owners whose dogs were diagnosed with a congenital heart disease at the authors’ institution in a 1-year period received a questionnaire during the consult after cardiac auscultation but before performing an echocardiogram. The main study aims were to reveal whether the breeders had informed the new owners about the presence of a murmur before purchasing the puppy, and whether the owners would have still chosen to buy that specific puppy if they had known about a potentially present congenital heart disease. **Results**: Of the 60 interviewed owners, 72% would have refrained from buying the puppy if they had known about the presence of a congenital heart disease. However, only 23% of them were informed about the presence of a cardiac murmur before purchase. **Conclusions**: Most owners would have chosen to buy a healthy puppy without a heart disease if they had been informed.

## 1. Introduction

Congenital heart diseases are not rare findings in dogs, and they could have high morbidity and mortality associations later in life when an anomaly is severe [1,2,3,4,5,6,7,8,9,10,11,12,13,14,15,16,17,18,19,20,21,22,23]. Cardiac auscultation in puppies is of high importance for early identification of a murmur, which can be the only finding suggestive of a congenital heart disease [2,3]. Generally, the first veterinary health check takes place at the age of 6 weeks, coinciding with the first vaccination. It is of the utmost importance to reveal the cause of a suspected or clearly pathological heart murmur before the puppy is sold to a new owner. Selling a puppy with a severe congenital heart disease can have remarkable emotional and financial consequences for the new owner and the breeder, as well as legal sequelae for the breeder and the breeder’s veterinarian [4,5].

For a first opinion veterinary practitioner, distinguishing a nonpathological from a pathological murmur by auscultation alone can be challenging [2,3]. Any heart murmur of greater intensity score than 2 out of 6 (according to Levine’s scale) should be considered pathological until it is proven otherwise [2]. Consequently, each puppy with a moderate-to-loud murmur should ideally be referred to a veterinary cardiology specialist for diagnostic workup as soon as possible, even if the puppy is free of clinical signs [2,3].

A recent study showed that the median time interval between first murmur documentation and the examination by a veterinary cardiology specialist was 3.3 months [3]. This means that most puppies were referred for evaluation of a cardiac murmur to a cardiologist after the puppy was already sold to a new owner, as breeders typically sell their puppies between 8 and 10 weeks of age. In the cited study, only 10% of dogs were referred for murmur evaluation to a cardiologist by the breeders’ veterinarians [3]. Additionally, 75% of the breeders’ veterinarians failed to record the presence of a cardiac murmur in the pet’s passport, which is the only available legal written document to the new owner at the time of purchase [3].

The primary aim of the present study was to reveal whether the new owners of puppies with confirmed congenital heart disease were informed about the presence of a cardiac murmur by the breeder before the day of purchase and whether they would have still bought that specific puppy if they had known about the potential presence of the congenital heart disease. A second aim of the study was to investigate what the referring veterinarians told the owners about the meaning of a cardiac murmur before referral.

## 2. Materials and Methods

The study design was a prospective questionnaire-based case series. Questionnaires were given to owners, who owned an asymptomatic puppy of less than 6 months of age with a suspected pathological murmur at referral, or a dog of any age where the cardiology specialist found a congenital heart disease as the cause of the murmur on the day when the questionnaire was filled in. All dogs had to be referred for evaluation of a cardiac murmur to the cardiology service of the authors’ institution between March 2021 and March 2022. No questionnaire was given to those owners, who were the breeders of the presented puppy, and to those owners, whose puppies were diagnosed with an innocent murmur by the cardiologist. Participation of all owners took place on a voluntary basis and all owners signed an informed consent agreeing that their response can anonymously be used for research purposes.

Printed one-page questionnaires were handed out to the owners during their visit to the authors’ institution after the cardiology specialist performed a physical examination. Owners had the possibility to fill in the questionnaire in the waiting room while the cardiologist performed the echocardiography on their dogs. The questionnaire consisted of 9 questions, both multiple choice and short-answer open questions.

In addition to the questionnaires, information from the health certificate section of the pet passports were collected. The dates of veterinary health checks and the auscultation findings of the first opinion veterinarians (i.e., the breeder’s and the new owner’s veterinarians) regarding the presence of a cardiac murmur were collected from the puppies’ pet passports and/or medical records filed by the referring veterinarians.

The following pieces of information were collected: (1) the age of the dog at the visit to the cardiology service of the authors’ institution, (2) the age of the dog when the owners were informed about the presence of a cardiac murmur, (3) the source of information about the presence of the murmur, i.e., the breeder, the pet’s passport or the referring veterinarian, (4) the age of the dog at the first written documentation of the murmur in the health certificate section in the pet’s passport, (5) the age of the dog at the first written documentation of the murmur in the medical record and/or in the referring letter of the referring veterinarian, (6) the owners’ statement whether they would have chosen to buy this specific puppy, if they had known about its congenital heart disease, (7) whether the referring veterinarian communicated to the owner about the murmur as a sign of a potentially or certainly serious underlying heart disease, (8) the advised urgency of further investigation (i.e., referral) of the murmur by the referring veterinarian, (9) whether the referring veterinarian advised to wait with the referral to a cardiologist; if yes, for how long and what the reason for waiting was, (10) the actual action taken by the owners and their motivation, (11) whose idea it was to visit a veterinary cardiologist (i.e., the owner, the referring veterinarian or the breeder), (12) the age of the dog at the visit to a non-cardiologist veterinarian for an echocardiographic examination, if this took place at all before referral to the cardiologist. Owners who brought their self-bred puppies with a congenital heart disease to the cardiologist (i.e., breeders) did not receive a questionnaire.

At the authors’ institution, all dogs underwent a physical and echocardiographic examination by a veterinary cardiology specialist after history-taking. The echocardiogram was carried out on unsedated, manually restrained dogs in left and right lateral recumbency. The examination consisted of two-dimensional, M-mode, color and spectral (pulsed and continuous wave) Doppler modes from the standard left and right parasternal, as well as subcostal views [24,25].

### Statistical Analysis

Descriptive statistical analysis was performed using commercial statistical software (IBM^®^ SPSS^®^ Statistics 28.0.1.0, IBM Corporation, New York, NY, USA). Normality of continuous variables was assessed using the Shapiro–Wilk test. Equality of variance was assessed using Levene’s test. When data were not normally distributed, a log-transformation was performed. Non-normally distributed data are presented as median and range. The outcome of interest was the time interval between first murmur detection and first visit to a veterinary cardiologist. The modified *t*-test was used to compare the intervals between breeder or veterinarian as sources of information about the cardiac murmur in a dog. ANOVA followed by the Tukey post hoc test was used to compare the intervals between three groups of veterinary assessment of cardiac murmurs. The modified *t*-test was used to compare the intervals between veterinary advice to wait or seek immediate further investigation. Students’ *t*-test was used to compare the intervals between whether an echocardiographic examination was performed by a non-cardiologist or not. The chi-square test was used to calculate a correlation between veterinary advice and owner action. A two-sided *p*-value of <0.05 was considered statistically significant.

## 3. Results

### 3.1. Animals and Owners

In the one-year study period, 77 dogs were diagnosed with an innocent murmur or a congenital heart disease by the cardiology service at the authors’ institution. Of these 77 owners, 61 owners received a questionnaire, and the results of 60 questionnaires were analyzed. The reasons for not including the remaining 17 cases were: 1 completed questionnaire was lost, 4 dogs were diagnosed with an innocent cardiac murmur, and 12 dogs (15%) were referred for murmur evaluation while they were in the breeder’s possession. The median age of dogs at the authors’ institution was 8.3 months (range 52 days–10 years and 7 months). The median time interval between first detection of a heart murmur and the visit to the author’s institution was 2.2 months (range 4 days–10 years and 5 months).

Of the 77 dogs, 73 dogs (95%) were diagnosed with a pathological murmur and 4 dogs (5%) had an innocent murmur. In 53 of these 73 dogs (73%) a single congenital heart disease was found (Table 1), whereas in 10 dogs (14%) two, in 8 dogs (11%) three and in 2 dogs (3%) four unrelated cardiac anomalies were seen with echocardiography.

The murmur intensity was assessed and documented by four veterinary cardiology specialists at the authors’ institution.

All four dogs with an innocent murmur had a soft murmur with an intensity of 1 out of 6. Three of the seventy-three included dogs (4%) with a pathological murmur had a murmur intensity of 1 out of 6, four dogs (5%) had a murmur intensity of 2 out of 6, thirteen dogs (18%) had a murmur intensity of 3 out of 6, seventeen dogs (23%) had a murmur intensity of 4 out of 6, twenty-six dogs (36%) had a murmur intensity of 5 out of 6, and ten dogs (14%) had a murmur intensity of 6 out of 6, as judged by the attending cardiologist.

### 3.2. Source of Information about the Presence of a Murmur

All owners completed the questionnaire. One questionnaire got lost.

The median age of the 60 dogs was 2.5 months (range 41 days–7 years and 2 months) when their owners were first informed about the presence of a cardiac murmur. Only 13 out of the 60 owners (22%) were informed by the breeder about the presence of a murmur, and one additional owner was informed by the previous owner. All other owners (77%) were informed by their referring first opinion veterinarian after they bought their dogs. Though two owners stated that they were informed by their first opinion veterinarian about the presence of a cardiac murmur for the first time, the murmur had already been recorded in the health certificate section of the pet’s passport with a date before the purchase of the puppy. These two owners were not verbally informed about the presence of a murmur by the breeder.

### 3.3. Owners’ Statement on Buying a Puppy with a Potential Congenital Heart Disease

Forty-three out of sixty owners (72%) stated that they would have refrained from buying their dog if they had known about the presence of a congenital heart disease. Sixteen owners (27%) stated that they would have still bought their dog despite the presence of a congenital heart disease. One owner was in doubt, depending on the severity and long-term prognosis of the heart disease, about which information was unavailable when the questionnaire was completed.

### 3.4. Breeder’s Interpretation of the Murmur

Seven of the fourteen owners who were informed by the breeder or the previous owner about the presence of a cardiac murmur were told that the murmur was innocent. All these seven dogs had a congenital heart disease (Table 2). The murmur intensity of these seven dogs was at least moderate, according to the interpretation of the attending cardiologist. The other seven owners were told that the murmur could be a sign of a potentially serious heart disease.

### 3.5. Referring Veterinarians’ Interpretation of the Murmurs and Their Recommendation

Forty-three of the sixty referring veterinarians (73%) judged the cardiac murmur to be potentially serious. Nine veterinarians (15%) judged the murmur as certainly serious, and seven veterinarians (12%) judged the murmur as probably innocent. Their judgement in relation to the final diagnosis is shown in Table 2.

The referring veterinarians’ assessment had an influence on the time interval between first murmur detection and visit to a veterinary cardiologist. The median time interval was 54 days (range 4–444 days) when the referring veterinarian judged the murmur to be “certainly serious”, significantly shorter than the median time interval of 159 days (range 38–3790 days), when the murmur was judged to be “probably innocent” (*p* = 0.028). There was no significant difference in the time interval between the other groups.

Forty-five of the sixty referring veterinarians (75%) recommended immediate further investigation of the murmur and fifteen referring veterinarians (25%) recommended waiting with the referral. This advice significantly influenced the time interval between first murmur detection and visit to the veterinary cardiologist (*p* ≤ 0.001). When the referring veterinarian recommended immediate further investigation, the median interval between first murmur detection and visit to a veterinary cardiology specialist was 57 days (2 months, range 4–697 days). When the referring veterinarian recommended them to wait, this median time interval was 207 days (7 months, range 38–3790 days).

Four of the fifteen veterinarians who advised waiting upon hearing a murmur at the first health check at the age of 9 weeks, recommended to wait until the next health check, which was scheduled at the age of 12 weeks. If the murmur was still present at 12 weeks of age, further investigation was recommended. One veterinarian heard the murmur at approximately 5 months of age in an imported puppy and scheduled an additional health check after 2 weeks. One veterinarian of a breeder recommended waiting until the 9-week vaccination and health check, at which moment the new owners’ veterinarian recommended further investigation. Two veterinarians recommended waiting until there was an increase or decrease in the murmur intensity, and another two veterinarians recommended waiting until the development of clinical signs. Two veterinarians recommended further investigation of the cardiac murmur only prior to general anesthesia before neutering. One veterinarian recommended waiting until 1 year of age, and one until the owner had time to get the murmur investigated. In one dog, a soft murmur was heard at the age of 12 weeks, after which it was not detected again until 8 months of age. At this point, the veterinarian advised immediate further investigation. One veterinarian performed an echocardiogram at 9 weeks of age and found a mild aortic stenosis, and subsequently recommended a recheck after 3 months. This recheck was done at the authors’ institution, where a moderate aortic stenosis was diagnosed.

### 3.6. Owners’ Reasons for Waiting with Referral and Its Duration

Ten of the fifteen owners (66%), whose veterinarian recommended to wait with further murmur investigation, followed this advice. The remaining five owners (33%) decided to have their dogs’ cardiac murmur investigated immediately.

Five out of forty-five owners (11%), whose veterinarian recommended immediate referral, decided to wait with further investigation. The owners reported various reasons for delaying further investigation. Two owners hoped that the murmur would spontaneously disappear, of which one did not know that a cardiac murmur could be a sign of a congenital heart disease, which might have negative consequences among others breeding with the dog. One owner decided to wait until the development of clinical signs. One owner got a second opinion, where the cardiac murmur was judged to be much softer than the first veterinarian had described; therefore, the advice changed from seeking further investigation immediately to letting an echocardiogram be performed by a non-cardiologist at 6 months of age. The last owner had various reasons for postponing further investigation, including a holiday and financial issues. The remaining 40 owners followed their veterinarians’ advice to further investigate the cardiac murmur as soon as possible.

The referring veterinarians’ advice on whether to wait or not with the referral had a significant influence on the owners’ decision (*p* ≤ 0.001). Owners were six times (95% confidence interval [CI] 2.4–14.8) more likely to wait with further investigation when their veterinarian advised them to do so. Owners were 2.6 times (CI 1.3–5.5) less likely to wait with further investigation when their veterinarian advised them to seek immediate further investigation.

### 3.7. Echocardiography by Non-Cardiologist before Referral to a Cardiology Specialist

Forty-four out of seventy-seven dogs (57%) were first referred for echocardiography to a non-cardiologist. Fifty out of sixty owners (83%) reported that their veterinarian recommended visiting a veterinary cardiology specialist for further investigation of the cardiac murmur, either after murmur detection or after anomalies were found at the echocardiography performed by a non-cardiologist. The median age of these dogs at the first echocardiography was 172 days (5.7 months, range 69 days–4 years and 6 months). Echocardiography by a non-cardiologist before visiting a cardiologist did not significantly lengthen the time interval between the first murmur detection and the visit to a veterinary cardiologist (*p* = 0.19). The median time interval between echocardiography by a non-cardiologist and the visit to the veterinary cardiology specialist was 14 days (range 3 days–10 months and 23 days). Thirty-nine of the forty-four dogs (89%) were presented to a veterinary cardiology specialist within 40 days after their first echocardiogram elsewhere.

## 4. Discussion

Only 22% of new owners of a dog with a later confirmed congenital heart disease were informed about the presence of a cardiac murmur by the breeder. Whether or not the breeders knew about the heart murmur in the remaining puppies is unknown. Consequently, 78% of the new owners bought a puppy from a breeder with a congenital heart disease, without any suspicion that a health issue might have been present. Diagnosing most congenital cardiac anomalies should be possible when the puppy is still in the breeder’s possession, since the cardiac murmur of the most common and clinically relevant congenital cardiac anomalies, such as the left-to-right shunting patent ductus arteriosus and severe pulmonic stenosis, are typically loud and are audible from birth [8,9,10,11,12,13,14,15,16]. Whether the cardiac murmur was either missed by the breeder’s veterinarian, or the breeder’s veterinarian failed to record the murmur in the pet’s passport and/or communicate it to the breeder, and/or the breeder intentionally or unintentionally failed to inform the new owner about the presence of a cardiac murmur remains unknown.

In our study at least two factors resulted in delayed referral. The referring veterinarians’ advice significantly influenced the owners’ decision on whether to wait or seek immediate further investigation. The cardiology service of the authors’ institution could potentially be a contributing factor too because of the availability of appointments. However, the service has a policy that puppy owners get an appointment offered within a week. Our findings suggest a high level of owner trust in their veterinarians, and the owners seem to follow their veterinarians’ advice strictly on this subject, as was found in a previously published report [26]. The delay in the referral of a dog with a murmur in the present study was significantly longer when the cardiac murmur was assessed to be “probably innocent”, as opposed to “certainly serious”. All veterinarians who judged the murmur to be “probably innocent” advised the owners to wait with further investigation. In contrast, all veterinarians who judged the murmur to be “certainly serious” advised the owners to seek immediate further investigation. High owner concern about their pet’s disease has been reported to increase owners’ compliance with veterinary advice. One study found that a greater number of pet owners with cardiac diseases believed that the condition negatively impacted their pets’ quality of life, when compared to other organ dysfunctions [27]. We believe that it is important that veterinarians explain to owners that a cardiac murmur can be a sign of a severe congenital cardiac anomaly, which does not necessarily cause noticeable problems at a young age, but it may lead to morbidity and mortality months to years later. It is essential that first opinion veterinarians openly share the uncertainties and limitations about the value of physical examination in diagnosing a congenital heart disease in puppies with a murmur when they explain their auscultation findings to the owners. Our study showed that a quarter of the referring veterinarians purposefully recommended waiting with further diagnostics of a puppy with a murmur. However, because of concerns and uncertainty, a third of these owners with this advice did make an appointment at the cardiologist without any delay. We believe that recommending immediate referral to a cardiology specialist to every owner of a pup with a moderate-to-high intensity cardiac murmur would be the best practice. In this case, the owner can still make their own judgement and decide to postpone or not at all to go for a consultation to a cardiologist. As a consequence, the breeder can also be spared from reputational damage and financial and legal consequences if the presence of a congenital heart disease is known before a puppy is offered for sale or adoption.

The most common clinically relevant congenital heart diseases cause a loud murmur from birth [6,7,8,9,10,11,12,13,14,15,16]. Similarly to previous studies, left-to-right shunting patent ductus arteriosus, pulmonic stenosis, and aortic stenosis were the three most common causes of a cardiac murmur in the present study [1,3]. Of these three most prevalent congenital heart diseases, both patent ductus arteriosus and pulmonic stenosis are effectively treatable conditions [8,9,10,11,12,13,14,15,16]. For both diseases, the younger the dog when it undergoes catheter-based interventional or surgical treatment, the greater the chance that the disease can be cured, or the long-term prognosis can be markedly improved [8,9,10,11,12,13,14,15,16]. When clinical signs of heart disease are present before surgery, the long-term prognosis is less favorable [8,9,10,11,12,13,14,15,16]. Furthermore, in full-grown large and giant breed dogs with pulmonic stenosis, a technically more challenging large-diameter balloon valvuloplasty or the pricier double-balloon technique might be necessary for effective dilatation of the large vessels [16]. Therefore, the shortest possible delay from the first murmur detection to the referral to a veterinary cardiology specialist is desirable. The best practice would be that no puppies with a suspected or confirmed congenital heart disease were sold to new owners, unless the potential owners are fully informed about the possible consequences of such a condition, and they specifically choose buying a pup with a heart disease. Not all serious congenital heart diseases cause a loud murmur at 6 weeks of age. Because subaortic stenosis is more of a developmental than a congenital heart disease, it is possible that the murmur caused by this condition is not yet audible at the first health check at 6 weeks of age [17,18,19,20]. It is also important to note that some clinically relevant congenital heart diseases cause no or only a soft murmur, which makes the index of suspicion of a severe heart disease low based on auscultation alone. Examples of these congenital heart diseases in dogs include atrial septal defect, right-to-left shunting patent ductus arteriosus, and cor triatriatum dexter [21,22,23].

More than half (57%) of the dogs underwent an echocardiographic examination by a non-cardiologist before referral to a veterinary cardiology specialist. Considerations for performing non-specialized echocardiography first could have some advantages, such as lower examination costs, shorter travel distance, and possibly quicker or more flexible appointment options. However, this practice could cause unnecessary delay in interventional or surgical therapy. Interestingly, in the present study this extra diagnostic step did not significantly lengthen the interval between first murmur detection and visit to a veterinary cardiologist, compared to the owners, who had not gone to a non-cardiologist echocardiogram. The relatively quicker referral to a cardiologist after an echocardiogram elsewhere might have been due to concerning or unclear echocardiographic findings. The number of dogs that did not get referred to a cardiologist after an echocardiogram was performed by a non-cardiologist remains unknown.

Only 27% of the interviewed owners stated that they would have chosen the same dog even if they had known about the presence of a congenital anomaly. However, 72% of the owners stated that they would have refrained from buying that specific puppy if they had known about the potential presence of a congenital heart disease. This finding highlights the emotional and/or financial burden, as well as the frustration of buying a puppy with a hidden congenital heart disease [4,5]. Because pets are considered as family members, even subclinical diseases can have a major negative psychological impact on their owners [28,29]. Why the minority of owners would still have chosen to buy their puppy if they had been informed about the presence of a potential congenital heart disease can be partly explained by the results of a recent study conducted in Denmark [30]. Owners of four different dog breeds reacted differently to health issues of their pets: owners of Chihuahuas and Cavalier King Charles Spaniels showed a closer owner–dog relationship with increasing health issues, as opposed to owners of French bulldogs [30].

The present study has several limitations. The sample size is relatively small, and the data were collected at a single tertiary academic referral centre in a West European country. Cultural differences in other countries or continents might have resulted in a different percentage of owners who would have chosen to buy or adopt a dog with a known congenital heart disease. Another limitation is that the owners filled in the questionnaire before they knew the type and the severity of their dog’s heart disease and the associated prognosis. For logistic and emotional reasons, the authors felt it more appropriate to let the owners complete the questionnaire before the definitive diagnosis was known to them. However, the definitive diagnosis with prognostic information and available therapeutic options (also including risks and costs) might have changed the owners’ opinion in either direction. The original information the owners heard from the breeder and the referring veterinarian about the meaning of the murmur might have been different from the one the owners reported in the questionnaire as it was subject to the owners’ personal interpretation and possibly colored with emotions. What, when, and how exactly the breeders and referring veterinarians told the owners about the meaning of a heart murmur remains unknown. Using Levine’s scale for describing murmur intensity is also a limitation because of its subjective nature, e.g., differentiating 1 from 2 and 5 from 6 out of 6 murmurs. Using a recently proposed 4-scale murmur grading system would have still offered a clinically meaningful murmur characterization [31].

## 5. Conclusions

Although less than a quarter of the interviewed owners were informed about the presence of a cardiac murmur of their puppy before the day of purchase, almost three quarters of them stated that they would have refrained from buying the dog if they had known about the presence of a congenital heart disease.

Three quarters of the referring veterinarians recommended immediate referral of the puppy when they detected a cardiac murmur at the veterinary health check. The significantly shorter time interval between the first murmur detection and the actual visit to a veterinary cardiology specialist emphasizes the high level of trust in veterinary advice on the owner’s decision. Referral for an echocardiography by a non-cardiologist before referral to a cardiologist took place in more than half of the cases, but this did not significantly delay the referral to a veterinary cardiologist.

## Figures and Tables

**Table 1:** Type of congenital heart diseases and the associated murmur intensity found in the 77 dogs that were presented to the authors’ cardiology service in the study period.

Cause of Murmur	Number of Dogs	Murmur Intensity (Median, Scale 6)
**Isolated congenital anomalies**	**53**	
Patent ductus arteriosus *	17	5
Pulmonic stenosis	15	
severe	8	4.5
moderate	6	4
mild	1	5
Aortic stenosis	10	
severe	2	4.5
moderate	4	3.5
mild	4	2
Ventricular septal defect *	5	4
Tricuspid valve dysplasia	3	4
Mitral valve dysplasia	2	2
Double chambered right ventricle	1	5
**Multiple unrelated congenital anomalies**	**20**	
Two anomalies	10	5
Three anomalies	8	4
Four anomalies	2	5.5
**Innocent heart murmurs**	**4**	1

* all left-to-right shunting.

**Table 2:** The referring veterinarians’ assessment of the meaning of the murmur in 73 dogs with a congenital heart disease in relation to the final diagnosis and murmur intensity, as assessed by the cardiology specialist.

Referring Veterinarian’s Judgement of Murmur	Median Murmur Intensity as Rated by the Cardiologist	Diagnosis	Number of Dogs
Probably innocent (n = 7)	5/6	Patent ductus arteriosus	2
		Multiple (2) anomalies	3
		Aortic stenosis	
		moderate	1
		Pulmonic stenosis	
		severe	1
Certainly serious (n = 12)	4.5/6	Patent ductus arteriosus	4
		Aortic stenosis	
		mild	1
		moderate	1
		Multiple (3 to 4) anomalies	3
		Tricuspid valve dysplasia	2
		Pulmonic stenosis	
		severe	1
Potentially serious (n = 40)	5/6	Patent ductus arteriosus	7
		Ventricular septal defect	4
		Aortic stenosis	
		mild	2
		moderate	1
		severe	2
		Pulmonic stenosis	
		mild	1
		moderate	5
		severe	5
		Multiple (2–4) anomalies	10
		Tricuspid valve dysplasia	1
		Mitral valve dysplasia	1
		Double chambered right ventricle	1

## Data Availability

The data that support the findings of this study are available from the corresponding author upon reasonable request.

## Abbreviations

CI = confidence interval
